# Myval Transcatheter Heart Valve: The Future of Transcatheter Valve Replacement and Significance in Current Timeline

**DOI:** 10.3390/jcm13226857

**Published:** 2024-11-14

**Authors:** Teoman Kilic, Senol Coskun, Didar Mirzamidinov, Irem Yilmaz, Sadan Yavuz, Tayfun Sahin

**Affiliations:** 1 Structural Heart Interventions Unit, Department of Cardiology, Kocaeli University School of Medicine, Kocaeli 41380, Turkey; didar.mirzamidinov@kocaeli.edu.tr (D.M.); irem.yilmaz@kocaeli.edu.tr (I.Y.); tayfun.sahin@kocaeli.edu.tr (T.S.); 2 Faculty of Health Sciences, Kocaeli Health and Technology University, Kocaeli 41275, Turkey; senol.coskun@kocaelisaglik.edu.tr; 3 Department of Cardiovascular Surgery, Kocaeli University School of Medicine, Kocaeli 41380, Turkey; sadan.yavuz@kocaeli.edu.tr

**Keywords:** Myval, safety, transcatheter aortic valve implantation, transcatheter heart valve, valve-in-valve implantation

## Abstract

The Myval is a balloon-expandable transcatheter heart valve (THV) developed by Meril Life Sciences Pvt. Ltd. (Vapi, Gujarat, India) that has an innovative operator-friendly design that aids in improving deliverability and features precise deployment. Various clinical studies demonstrate its effectiveness and safety, making it a promising choice in valvular interventions. Myval has been successfully utilized as a transcatheter aortic valve implantation (TAVI) device in cases with conduction disturbances, bicuspid aortic valve anatomy, non-calcified aortic regurgitation, dysfunctional stenosed right ventricular outflow tract (RVOT) conduits, pulmonary valve replacement, mitral valve replacement, and valve-in-valve and valve-in-ring implantation procedures. Myval’s diverse sizes are also of key importance in complex cases of large annuli and complex anatomy. Further long-term studies are needed to consolidate these results. Its introduction signifies a significant advancement in cardiology, aiming to enhance patient outcomes and quality of life. In the present review, we provide an update on new-generation Myval THV series and review the available clinical data published to date with an emphasis on diverse use in specific clinical scenarios.

## 1. Introduction

Transcatheter aortic valve implantation (TAVI), pioneered by Cribier in 2002, has become an effective alternative to surgical aortic valve replacement (SAVR) for patients with symptomatic aortic stenosis (AS) [1,2,3]. Aortic valve replacement (AVR) is the Class I recommendation for patients with stage D1 AS and symptoms of exertional dyspnea, heart failure, angina, syncope, or presyncope by history or on exercise testing. It is also recommended for patients with severe asymptomatic AS and left ventricular ejection fraction (LVEF) below 50% (stage C2) [2]. The indications for AVR and recommendations for SAVR vs. TAVI are shown in Table 1 and Table 2. Complication rates, including mortality, strokes, and vascular problems, have significantly decreased over time owing to technological advancements and expanding indications, all without raising major concerns about the durability of transcatheter heart valves (THVs) [4,5,6]. The current focus lies on a tailored approach, aiming to offer each patient the most suitable THV based on their specific anatomy and ensuring optimal hemodynamic performances after the index procedure and potential for repeatability in patients with increased longevity [7]. The indisputable success of TAVI in patients with severe and high risk quickly altered the treatment of aortic stenosis, prompting studies in patients with intermediate and eventually reduced surgical risks [8].

Compared to SAVR, TAVI is linked to a 2-year decrease in all-cause mortality and stroke, regardless of the kind of THV system or baseline surgical risk {hazard ratio (HR) 0.88 [95% confidence interval (CI): 0.78–0.99], *p* = 0.030} [9]. Similarly, in a study done by Blankenberg et al., with severe AS at low or intermediate surgical risk patients, in terms of all-cause mortality or stroke at one year, TAVI was non-inferior to SAVR; the Kaplan–Meier estimate of the primary outcome at one year was 5.4% vs. 10.0% (HR for death or stroke: 0.53; 95% CI, 0.35–0.79; noninferiority *p* < 0.001) [10]. In another nationwide real-world data analysis in Korea, TAVI with current-generation devices showed significantly lower 1-year mortality compared to SAVR in severe AS patients. TAVI was associated with reduced 1-year mortality (HR: 0.55; 95% CI: 0.42–0.70; *p* < 0.001) [11].

Two major types of TAVR are available in the current market: balloon-expandable valves (BEVs) and self-expanding valves (SEVs). In the study done by Senguttuvan et al., data from six studies with 2935 patients (BEV: 1439 and SEV: 1496) were assessed. The study demonstrated that when compared to SEV, BEV is related with lower risk of all-cause mortality (2.2% vs. 4.5%; RR: 0.51; 95% CI: 0.31–0.82; *p* < 0.006) and cardiovascular mortality (2.5% vs. 4.3%; RR: 0.54; 95% CI: 0.32–0.90; *p* = 0.01) at 30 days. Implantation of >1 valve per procedure (0.78% vs. 5.11%; RR: 0.15; 95% CI: 0.07–0.31; *p* < 0.00001) and moderate/severe aortic regurgitation (AR)/paravalvular leak (PVL) (2.5% vs. 9.01%; RR: 0.3; 95% CI: 0.17–0.48); *p* < 0.00001) were lower in the BEV group [12]. In another recent propensity-matched analysis by Van Belle et al., the first coprimary outcome incidence was higher with the SEV group (19.8%) compared with the BEV group (11.9%; RR: 1.68 [95% CI, 1.46–1.91]; *p* < 0.0001). The SEV group had higher ≥ moderate paravalvular regurgitation (PVR) (15.5% vs. 8.3%; RR: 1.90 [95% CI, 1.63–2.22]; *p* < 0.0001) and in-hospital mortality (5.6% vs. 4.2%; RR: 1.34 [95% CI, 1.07–1.66]; *p* = 0.01). All-cause mortality occurred in 899 patients in the SEV group (2-year mortality, 29.8%) and in 801 patients treated with the BEV group (2-year mortality, 26.6%; HR: 1.17 [95% CI, 1.06–1.29]; *p* = 0.003) [13].

Meril Lifesciences Pvt. Ltd. (Vapi, India) has developed the Myval, a new-generation BE THV that features an innovative operator-friendly design that enhances deliverability and enables precise deployment. In 2018, Myval received approval from the Central Drugs Standard Control Organization (CDSCO) in India, and it was granted a CE mark by the European Economic Area in 2019. In the present review, we provide an update on new-generation Myval THV series and review available clinical data with an emphasis on diverse use in specific clinical scenarios.

## 2. Myval Transcatheter Heart Valve System

**Figure 1:**
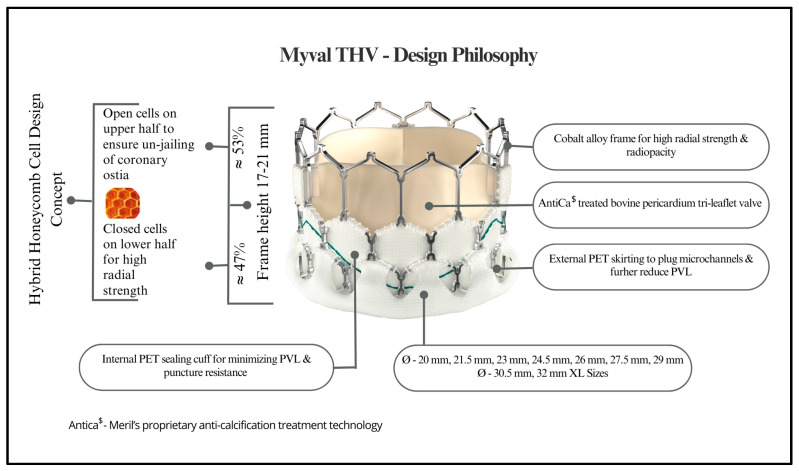
Structural overview and salient features of the Myval transcatheter heart valve.

The Myval consists of MP35N cobalt alloy-framed hexagons, configured in a hybrid honeycomb structure. The structure consists of open cells (53%) at the aortic end and closed cells (47%) at the ventricular end, which provide a greater annular radial force [14]. The alternative dark/light band-like pattern produced on crimping serves as reference markers for locating and deploying THV over the native annulus. A polyethylene terephthalate (PET) sealing cuff encases the lower “closed cell” section of the valve frame externally to reduce PVL as depicted in Figure 1. Myval currently offers a range of sizes: conventional sizes include 20 mm, 23 mm, 26 mm, and 29 mm; intermediate sizes are 21.5 mm, 24.5 mm, and 27.5 mm; and extra-large sizes are 30.5 mm and 32 mm. This variety allows clinicians to choose the most appropriate valve size for different native annulus shapes. The Navigator THV delivery system (Meril Life Sciences Pvt. Ltd., Vapi, India) is a specialized high-flex, over-the-wire balloon catheter delivery system designed for the administration of Myval. The distal end of this system can be flexed beyond 180 degrees while traversing the native valve and the aortic arch. At the distal end of the delivery system, a shallow, low-profile zone is formed by a balloon with two internal counter-opposing soft stoppers where the Myval THV is directly crimped. The procedure is safer, simpler, and more intuitive with the “deliver and implant” function considering there is no covering sheath, implying that the stoppers restrict the valve from dislodging during delivery. The dog bone-shaped balloon’s proximal and distal internal inflation ports stabilize the valve during inflation and deployment. The pre-mounted THV can pass through an expanding hydrophilic Python introducer sheath (Meril Life Sciences Pvt. Ltd., Vapi, India) with an internal diameter of 14 Fr, which expands temporarily to accommodate all sizes of crimped Myval [14]. The sheath design allows for complete valve retrieval from the patient in case the operator is not able to deploy the valve. Before Myval is deployed, the operator may choose to perform a balloon valvuloplasty on the native valve.

Myval is supplied through the native annulus, and the valve is accurately positioned using the dark and light bands as reference markers, ensuring that the second distal dense band is aligned with the annular plane. Post-implantation aorto-ventricular depth of 80:20 or 90:10 relative to the annulus will be attained [15].

### Myval Octacor

**Figure 2:**
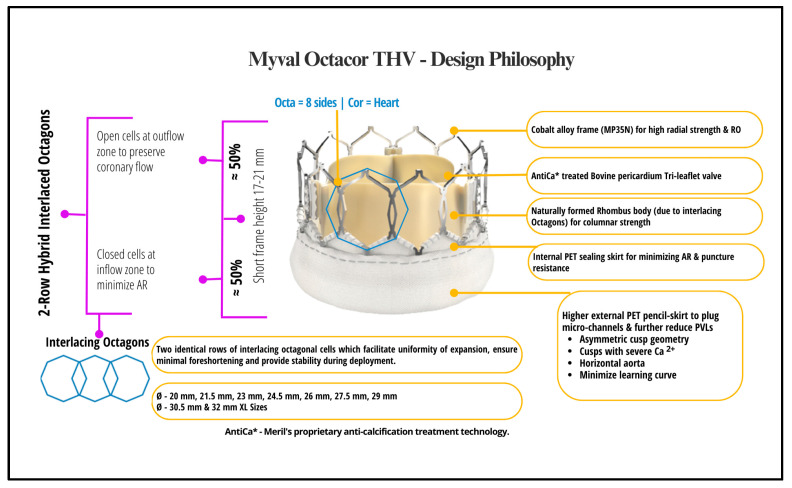
Design and key features of the new-generation Myval Octacor THV system.

Myval Octacor THV is the new iteration of the BE Myval THV. Compared to the previous Myval version, the new device design contains only two rows of similar octagonal cells [16]. In order to reduce the occurrence of PVL, oversizing BE THVs has been described as possible and advised within safe bounds. The median oversizing ratio, as determined by the Myval Octacor, was 8.2%, falling within the permitted range of less than 10% [17]. The propensity for PVL is minimized because the external skirt of the frame is up to 50% of the frame in the Myval Octacor THV (Figure 2). A landing zone marker towards the ventricular end of the Navigator Inception THV delivery system facilitates precise positioning of the Myval Octacor THV at the annulus [18]. Among BE valves, the Myval Octacor stands out as the only one equipped with a technique for commissural alignment [18]. Severe commissural misalignment can hinder coronary flow and access to the coronary ostia after TAVI and redo-TAVI, as well as causing early valve degradation. The OctaAlign technique is an advanced method for aligning the commissures of the Myval Octacor THV with the patient’s native aortic valve by aligning just one commissure towards the mirror image of mid-RCC in fluoroscopic view, which will deploy the valve anatomically towards the RCC-NCC commissure with minimal misalignment. This alignment reduces the risk of coronary artery obstruction, optimizes future coronary access after valve implantation, and minimizes complications in repeat procedures [19]. The proposed OctaAlign technique is easy to practice, does not require additional hardware or imaging software, is predictive and replicable, and ensures minimal commissural misalignment. Preliminary experience showed good results in the first 30 cases [19].

The next-generation Myval Octacor transcatheter heart valve (THV) was assessed by Jose et al. [16] for its safety and effectiveness in 123 patients suffering from severe symptomatic AS across 16 centers in India. The study showed that the Myval Octacor THV is both safe and effective for patients with severe AS. The rate of permanent pacemaker implantation (PPI) was found to be acceptable; however, some patients who required PPI had pre-existing conduction disturbances, including complete heart block, left bundle branch block (LBBB), right bundle branch block (RBBB), and atrial fibrillation.

The study by Elkoumy A et al. is the first of its kind to report the post-TAVI residual aortic regurgitation (AR) after real-world implantation of the newly designed BE Myval Octacor THV system and AR analyzed by validated quantitative video densitometry [17]. The study findings demonstrated a reduction in moderate or more AR, with a significant shift from mild to none or trace. Based on the qLVOT-AR results, the Myval Octacor THV exhibited a significant reduction in post-TAVI AR, comparable to the best new THV generations (either SE or BE) analyzed using the same technology [20]. The median depth of deployment was minimal (4.1 mm) but with a non-significant association between implantation depth and AR severity [17].

## 3. Pre-Clinical Evaluation of Myval

The development of the Myval THV began in 2015. Fatigue testing was conducted for 200 million cycles, which corresponds to approximately 5 years of bioprosthetic life for a patient, in accordance with the International Organization for Standardization (ISO) guidelines 5840-1:2021 and 5840-3:2021. The valves were also tested for endurance up to 400 million cycles (equivalent to 10 years of use) and 600 million cycles (15 years of use), yielding favorable results. Additionally, no device-related mortality was seen in animal studies during implantation of the Myval THV in a sheep (ovine) model using the innovative technique of external banding at the ascending aorta to simulate calcification and deployment of the Myval THV (either 20 or 21.5 mm) using a trans-carotid TAVI approach [21]. Eleven Myval THVs were implanted via the carotid approach. In total, two procedure-related deaths and premature deaths for each were reported. There was no substantial regurgitation, calcification, thrombi, or vegetation in any of the seven valves that had finished follow-up at 6 months. An ejection fraction of 53.3 ± 6% and a mean pressure gradient of 21.9 ± 11 mm Hg were recorded. The biocompatibility and hemodynamic performance of the Myval THV were optimal [21].

## 4. MyVal-1 First In-Human Study

According to the guidelines set forth by Indian regulatory authorities, this prospective, multicenter, single-arm, open-label study assessed the safety and efficacy of the Myval THV in 30 Indian patients who had severe symptomatic native AS and an ejection fraction ≥ 20% who were either high- or intermediate-risk for SAVR (CTRI/2016/11/007512) [14]. Survival at the 12-month follow-up was the primary safety endpoint. The efficacy endpoints included improvement in the effective orifice area (EOA) at echocardiography, the New York Heart Association (NYHA) functional classification, quality of life as determined by the Kansas City Cardiomyopathy Questionnaire (KCCQ), a 6 min walk test from baseline, and freedom from major adverse cardiac, cerebrovascular, and renal events (MACCRE) at the 12-month follow-up. Post-procedure clinical echocardiography was performed at 1 month, 6 months, and 12 months. Overall, the mean age of all patients was 75.5 ± 6.7 years. The mean STS Risk score was 6.4 ± 1.8, and 70% of patients were in NYHA functional class III/IV. Femoral access route was utilized in all procedures. Complete 100% device success was observed. Cumulative all-cause mortality rates at 1, 6, and 12 months were 3.3%, 6.7%, and 13.3%, respectively. Major vascular complications were reported in two patients (6.7%) and non-disabling stroke in one patient. Post-procedure, the EOA improved from 0.6 ± 0.2 cm^2^ to 1.7 ± 0.3 cm^2^, and the mean aortic valve gradient improved from 47.4 ± 8.8 mmHg to 8.0 ± 2.7 mmHg; these were sustained at 12 months. All patients had markedly enhanced quality of life and were in NYHA class I/II. More than a mild PVL was reported in no cases. No patient required a permanent pacemaker.

## 5. Early Clinical and Hemodynamic Outcomes of Myval in Comparison to Other THVs

In a retrospective study by Kawashima et al., the core lab analysis of quantitative video densitometric aortography was utilized to compare angiographic AR after TAVI among three BEVs: Myval (*n* = 108), Sapien 3 (*n* = 397), and Sapien XT (*n* = 239) [22]. The study found that the Myval THV had the lowest incidence of moderate to severe quantitative AR at 2.8%, compared to 8.3% for the Sapien 3 and 10.9% for the Sapien XT THV. The authors attributed this lower rate of AR to the external skirt design, which reduces PVL, as well as the availability of various intermediate and extra-large sizes. This design helps avoid the dangers associated with oversizing and undersizing, resulting in a more precise and optimal fit to the native annulus. Additionally, another retrospective analysis involving 1115 patients who underwent Myval implantation revealed that 42% of these patients used intermediate Myval sizes, addressing operators’ need for better-calibrated THV sizing in modern real-world practice [23].

Blinded echocardiographic investigation by Delgado-Arana et al. in 2022 demonstrated that the Myval BEV was safe, with a low rate of PPI and acceptable residual gradients and PVL rate. In the study, they compared a matched population of patients treated with Myval to patients treated with Sapien 3 (*n* = 103 each) [24]. Baseline characteristics were similar. Procedural success rate (Myval: 93.2%, Sapien-3: 94.2%; *p* = 0.219), 30-day mortality (Myval: 0.97%, Sapien-3: 2.9%; *p* = 0.625), clinical efficacy (4.9% vs. 12.6%, *p* = 0.057) and early safety (12.6 vs. 4.9%, *p* = 0.096) were comparable. In addition, in contrast to the Sapien 3 group, the Myval group required fewer PPIs (5.8% vs. 15.5%, *p* = 0.02) and had considerably lower mean gradients (*p* < 0.001). No significant differences were found in terms of ≥ moderate aortic regurgitation (0% for Myval, 1% for Sapien-3, *p* = 0.314). Investigators explained this by remarking that in almost 45% of the patients, intermediate Myval sizes were used, which may have produced a more appropriate fit.

Another retrospective analysis of 166 consecutive patients undergoing TAVR with either Myval (*n* = 58) or Evolut R SEV (*n* = 108) was done in the EVAL registry by Barki et al. [25] Primary objectives encompass evaluation of clinical efficacy (freedom from all-cause mortality, stroke, and cardiovascular hospitalization), echocardiographic performance, and PPI rates comparing the two THVs.

The Myval group showed significantly higher early device success in comparison to the Evolut R group (94.8% vs. 83.3%, *p* = 0.048). However, at 30 days and at 6 months, a significantly lower incidence of moderate or more PVL (6.9% vs. 19.8%, *p* = 0.039) and PPI (11% vs. 27.5%, *p* = 0.02) was observed in the Myval group. The all-cause mortality and disabling stroke rates were comparable in both groups.

A retrospective, single-center, propensity matched analysis (91 pairs) of Myval BE vs. Evolut SE valves revealed no significant difference in the rates of cardiac death (1% vs. 2%, *p* = 0.56), stroke (2% vs. 4%, *p* = 0.41), and myocardial infarction (1% vs. 3%, *p* = 0.31) [26]. The Myval group needed significantly less PPI (4% vs. 15%, *p* = 0.01). At 1 year, the stroke rate (7% vs. 5%, *p* = 0.76) and cardiac death (2% vs. 4%, *p* = 0.41) were comparable. Valve hemodynamics in both groups were remarkable, with a low rate of moderate-severe PVL.

The implantation of the Myval BEV was associated with improved valve hemodynamics, absence of moderate to severe PVL, and good safety outcomes at 6-month follow-up. In 2023, a study conducted by Halim et al. enrolled 120 consecutive patients who underwent TAVI using the Myval BEV. The clinical outcomes were evaluated at 30 days and 6 months post-procedure, following VARC-2 criteria [27]. An intermediate valve size was chosen for 51% of the patients. At the 6-month follow-up, cardiac death occurred in 0.8% of the patients, while the rate of all-cause death was 5.8%. Additionally, periprocedural stroke and the need for permanent pacemaker insertion were noted in 3.3% of patients each. Importantly, there were no complications related to access-site vascular issues or bleeding. Improvements in valve hemodynamics were observed, and no instances of moderate to severe PVL were reported at the 30-day mark.

Myval has also been successfully used in a failed sutureless Perceval bioprosthesis (Table 3) [28].

## 6. Myval Intermediate Follow-Up Outcomes

The SAPPHIRE prospective registry was conducted in two Italian centers between May 2020 and December 2020. The initial 100 patients (mean age 80.7 ± 7.7; STS 4.3 ± 3.3%) undergoing TAVR for severe AS were included [30]. Procedural and clinical outcomes were in alignment with VARC-3 criteria. Successful transfemoral implantation of the Myval THV was performed with 100% technical success and 99% device success. Vascular access complications were minor and managed by compression/balloon inflation. There were no instances of coronary occlusion and annular dissection or annular rupture; 5% of patients needed an in-hospital PPI. At one year, the rates of overall and cardiovascular mortality were 8% (CI 5–7%) and 5% (CI 2–5%), respectively; at two years, they were 12% (CI 9–14%) and 7% (CI 6–9%). PPI was required by 9% of the patients within 12 months, and no further PPI cases occurred afterwards. Between discharge and the 2-year follow-up, there were no myocardial infarction, renal failure, or cerebrovascular events. While there were no instances of structural valve deterioration, there was a persistent improvement in echocardiographic parameters. The Myval THV showed a promising safety/efficacy profile at 2-year follow-up.

Similar results were observed in a 2-year follow-up study by Moscarella et al. The Myval showed better clinical efficacy compared to the Evolut R, with a success rate of 86% versus 66% (hazard ratio: 2.62, 95% confidence interval: 2.2–5.1; *p* = 0.006). Additionally, there were fewer cardiac hospitalizations in the Myval group (3.4% compared to 13.9%, *p* = 0.03) [31]. No significant differences were found in cardiovascular mortality, all-cause mortality, or stroke rates. However, the proportion of patients with moderate or greater PVL was significantly lower in the Myval group (4% versus 22%, *p* = 0.008). The mean transvalvular gradient was significantly higher in the SEV group compared to the BEV group, measuring 9.5 ± 4.3 mmHg versus 6.9 ± 2.2 mmHg (*p* < 0.001).

In a recent multi-center, registry-based, observational study conducted by Kilic et al., 1-year and 2-year follow-up post-TAVI with Myval implantation was conducted in 207 consecutive degenerative severe AS patients [32]. Clinical and procedural outcomes were defined in alignment with VARC-3 criteria. Technical success was observed in 99% (204 patients), device success was observed in 91% (189 patients), early safety was observed in 78% (161 patients) and clinical efficacy was observed in 79% (163 patients). The death rate at 30-day was 7.7%; cardiovascular reasons were responsible for 3.4% of these deaths. At 1-year follow-up, all-cause and cardiovascular mortality rates were 9.7% and 4.3%, and at 2-year follow-up, 17.4% and 9.7%, respectively. Incidence of ≥moderate PVL at 30 days and 1-year and 2-year follow-ups were 3.4%, 4.3%, and 4.8%. A total of 11.1% of patients required a PPI at 30 days after implantation, while the cumulative rate of PPI at 2 years was 12.1%.

These favorable clinical and hemodynamic outcomes of Myval, which have been attributed to its unique design and intermediate valve sizes, are being studied in two current prospective, large, randomized trials: the LANDMARK trial [29] (non-inferiority trial over contemporary THVs—Sapien and Evolut series) and the COMPARE-TAVI trial.

## 7. The LANDMARK Trial

The LANDMARK trial was designed as a prospective, randomized, multinational, multicenter, open-label, non-inferiority trial ongoing with 768 patients randomized (1:1) to receive either the Myval (*n* = 384) or contemporary THV (*n* = 384)—either from the Sapien THV series (*n* = 192) or the Evolut THV series (*n* = 192) [29]. The primary combined safety and effectiveness endpoint at 30 days was a composite of VARC-3 defined endpoints: all-cause mortality, all stroke, bleeding (VARC type 3 and 4), acute kidney injury (stages 2, 3, and 4), major vascular complications, moderate or severe PVR, and conduction system disturbances resulting in a new PPI.

The LANDMARK trial proved that the Myval THV series was non-inferior to the contemporary (Sapien THVs and Evolut THVs) THV series at the 30-day follow-up in terms of primary composite endpoint (primary endpoint rate: 25% vs. 27%; risk difference (95% CI): −2.3% (NA to 3.8); P_non-inferiority_ < 0.0001).

The median age and STS score of patients were comparable. In the Myval arm, approximately 48% of patients were implanted with intermediate valve sizes (21.5, 24.5, and 27.5 mm). Post-dilatation rate was significantly higher in the contemporary valve group (21% vs. 10%; *p* < 0.0001). The contemporary group had higher rates of RF > 17% in final aortography (6% vs. 2%, *p* = 0.025). The mean pressure gradient and effective orifice area significantly improved from baseline to 30 days in both arms. The technical and device success was also comparable between Myval and contemporary valves [29].

## 8. Diverse Use of Myval THV in Specific Clinical Scenarios

**Figure 3:**
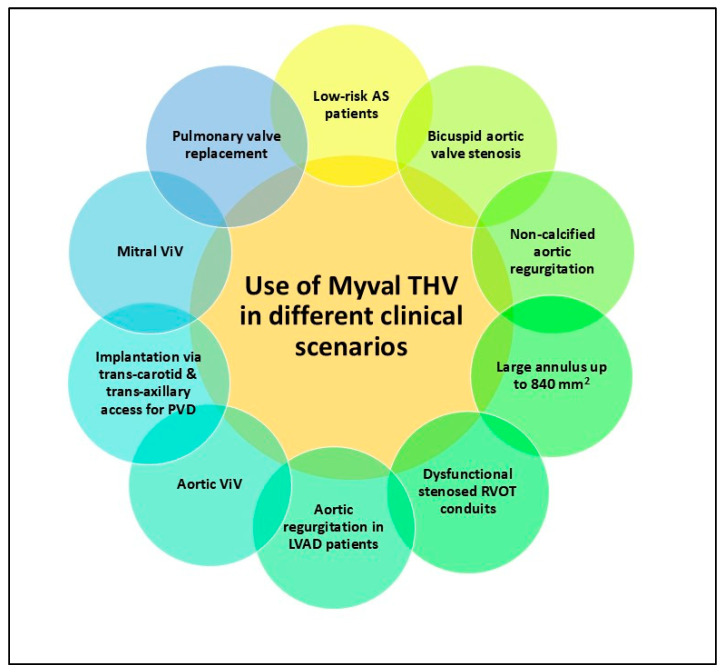
Diverse use of new-generation Myval THV system in different clinical scenarios.

The Myval THV has been used with reassuring safety and efficacy for a variety of indications, including calcific bicuspid aortic valve stenosis, valve-in-valve for degenerated bio-prosthetic surgical valves in aortic and mitral positions, and dysfunctional stenosed right ventricular outflow tract (RVOT) conduits (Figure 3).

### 8.1. Myval and Conduction Disturbances

The Myval group had the lowest rate of PPI (7.4%), as reported by Santos-Martinez et al. while analyzing conduction disturbances in 1131 consecutive patients of the Academic European Registry who underwent TAVI with any of the six THVs—Myval, Evolut, Sapien 3, Acurate, Portico, and Allegra [33]. Similar rates were observed amongst Sapien-3 (13.4%) and Acurate (9.1%). However, Evolut, Portico, and Allegra demonstrated significantly higher rates (18.5%, *p* = 0.003; 29.5%, *p* < 0.001 and 22%, *p* = 0.001, respectively).

### 8.2. Myval in Low-Risk as Patients

García-Gómez et al. conducted a retrospective study wherein they observed 100 patients who underwent TAVR using Myval at nine European centers and were at low risk for SAVR. These patients had low predicted operative mortality risk based on scores from the European System for Cardiac Operative Risk Evaluation (EuroSCORE-II) and the Society of Thoracic Surgeons (STS) [34]. Intermediate sizes were used in 39% of the cases. Procedural success of 99% was achieved. There were no cases of annulus rupture, valve embolization, coronary occlusion, or procedural death. No deaths were reported. The PPI rate was 8%. At 30-day follow-up, aortic valve area (0.7 ± 0.2 vs. 2.1 ± 0.6 cm^2^) and mean aortic valve gradient (43.4 ± 11.1 vs. 9.0 ± 3.7 mmHg) improved significantly (*p* < 0.001). Moderate AR occurred in 4%. Endpoints of early safety and clinical efficacy were 3% and 1%, respectively.

### 8.3. Myval in Bicuspid Aortic Valve Severe Stenosis

Nearly 50% of candidates for AVR have bicuspid aortic valves (BAVs) [35]. TAVI with the Myval THV in selected BAV anatomy was associated with favorable 1-year hemodynamic and clinical outcomes in a multicenter real-world experience in 62 patients as reported by Elkoumy et al. [36]. All-cause mortality was reported in 11.3%, cardiovascular hospitalization in 10.6%, all-stroke in 3.2%, PPI in 8.3%, and myocardial infarction in 1.6% of patients. Moderate AR was reported in only 2% while mild AR in 27%. Moderate (Stage II) hemodynamic deterioration was seen in 3 (6.4%) cases and severe (Stage III) hemodynamic deterioration in one (2.1%) case.

Amat-Santos et al. in 2023 published the TRITON study, a multicenter registry of patients with severe BAV stenosis treated with the BE THV (Myval and SAPIEN 3 Ultra, S3U) or SE THV: Evolut PRO+ (EP+) [37]. A total of 360 patients (age 76.6 ± 7.6 years, 71.9% males) were enrolled: Myval (122–33.9%), S3U (129–35.8%), and EP+ (109–30.3%). Tri-match analysis was done in order to reduce the influence of baseline variations. The 30-day device success was the study’s primary goal, while the individual and composite early safety components at 30 days were its secondary endpoints. The STS score average was 3.6 ± 1.9%. Device success at 30 days was higher with Myval at 100%, as compared to S3U (87.5%) and EP+ (81.3%). In patients with BAV stenosis considered unfit for surgery, Myval, S3U, and EP+ exhibited comparable safety profiles; however, BE Myval demonstrated superior gradients compared to S3U, and both BE devices presented lower residual AR than EP+. This indicates that when accounting for patient-specific risks, any of these devices may be chosen for optimal outcomes. Aortic dissection, annulus rupture, coronary artery blockage, and procedure mortality were not observed in any instances. This overall difference was mostly caused by S3U’s higher residual aortic gradients and EP+’s higher ≥moderate AR. The unadjusted pacemaker insertion rate showed no discernible variations.

### 8.4. Myval for Non-Calcified Aortic Regurgitation

The results of a recent meta-analysis of 11 trials, which included 911 patients receiving TAVI for NCAR, showed that the device success rate was 80.4%, that 7.4% of patients had ≥moderate AR, and that the 30-day mortality rate was 9.5%, with up to 3% of patients needing conversion to open surgery [38]. These rates compare unfavorably with the results of TAVI in AS. The Trilogy TAVI system (Jena Valve Technology, Irvine, CA, USA), a specially designed device for NCAR, does not yet support annuli with diameters greater than 27 mm [39,40]. The Myval BEV (Meril Life Sciences Pvt. Ltd.) offers the potential advantage of covering the largest range of annular areas (up to 840 mm^2^ at nominal inflation volume, 32.7 mm diameter). TAVR in NCAR is an off-label procedure.

In another multicenter, observational study, 113 consecutive patients with symptomatic severe NCAR undergoing TAVR with the Myval device were enrolled [41]. The mean STS score was 2.7 ± 1.7%. The mean EuroSCORE II was 3.48 ± 2.7%. The mean annular area was 638.6 ± 106.0 mm^2^, 59.3% of patients had aortic root dilatation, and 7.1% had bicuspid valves. The indicated range for extra-large sizes was surpassed in 2.6% of cases, and an additional volume (median 4 cc, up to 9 cc) was added in 92% of cases. In 95 patients (84.1%), the extra-large (XL) size was used, with a mean oversizing of 17.9 ± 11.0%. Technical success rate was 94.7%. Moreover, 8.9% residual ≥ moderate aortic regurgitation rate and a 22.2% PPI rate was reported. There were no cases of annular rupture, cardiac tamponade, or aortic dissection. However, in four patients (3.5%) valve embolization occurred (one antegrade and three ventricular); all had a tapered left ventricle outflow tract (*p* = 0.007). Mortality at 30 days and 1 year was 5.3% and 9.7%, respectively. Better survival was linked to technical success (97.1% vs. 72.7%; *p* = 0.012), and valve embolization was the primary cause of death (*p* = 0.047). For certain non-operable NCAR patients, Myval was a viable and safe choice that showed good midterm results and no effect of oversizing on device longevity.

### 8.5. Myval Sizing Using Annulus Perimeter

Perimeter sizing is a technique used in THV implantation procedures to determine the optimal valve size based on the annular perimeter rather than the area alone. This method aims to improve valve selection accuracy, especially in cases with irregular or asymmetric annuli, by providing a better contour match. A precise perimeter-based sizing algorithm was developed for the Myval THV, aimed at minimizing the annular oversizing to a maximum of 5% of its perimeter [42].

Myval BEV perimeter sizing results in good clinical outcomes, minimal PPI, and no significant PVL, as well as a major utilization of bigger valve sizes. With minimal PPI and no appreciable PVL, perimeter sizing with the Myval BEV results in a major utilization of larger valve sizes and positive clinical outcomes. In a prospective single-center study, Halim et al. assessed 60 patients with severe AS who had treatment with the Myval BEV [42]. A restricted oversizing of 3.7% ± 1.3% in relation to the annulus perimeter was employed while using perimeter sizing. Compared to the valve size determined by area sizing, a larger valve size was implanted in 33.3% of the patients. Clinical results were assessed 30 days and one year after TAVR. At 30 days, 2% and 3% of the patients, respectively, required a PPI and had a stroke. No cardiac death and moderate-severe PVL were reported at 30 days; however, at one year, cardiac death and stroke were observed in 3% and 8% of the patients, respectively.

### 8.6. Myval for Dysfunctional Stenosed RVOT Conduits

Transcatheter pulmonary valve implantation (TPVI) is a surgical alternative for the repair of faulty RVOT conduits in patients who have already undergone surgery. In a study conducted by Sivaprakasam et al., before the implantation of Myval in patients with stenosed dysfunctional conduits from the right ventricle to pulmonary artery (RV-PA), pre-stenting after initial computed tomography and balloon interrogation was done [43]. The size of Myval was selected based on the pre-stent’s final diameter. With a median age of 26 years, seven patients had stenosed RV-PA conduits implanted 5–17 years ago for tetralogy of Fallot (3), after a Ross surgery (2), for pulmonary stenosis correction (1), and after PA debanding (1). Pre-stenting reduced the gradient from 87.3 ± 31.7 mmHg to 12.7 ± 6.4 mmHg and increased the conduit diameter from 9.3 ± 2.8 mm to 20.8 ± 1.1 mm. There were no adverse effects associated with the valve, despite the fact that one patient required a second valve-in-valve implantation. The early results of TPVI with the Myval THV in pre-stented conduits are encouraging, since all patients had procedural success, and the mid-term results were acceptable.

Houeijeh et al. studied a case of a patient who had surgical fallot repair with chronic heart failure [44]. Investigations found severe biventricular dysfunction and enlargement due to chronic pulmonary regurgitation. The RVOT was tortuous and large with a diameter of 35 mm. Percutaneous pulmonary valve implantation (PPVI) with a 32 mm Myval THV over-sized to 35 mm, was performed post a challenging presenting, which demonstrated a favorable outcome.

### 8.7. TAVI for AR in Patients with Left Ventricular Assist Device (LVAD)

In the first year following implantation with a continuous-flow left ventricular assist device (LVAD), 25% to 30% of patients experience aortic regurgitation (AR), which is increasingly being identified as a contributing factor to the return of symptomatic heart failure (HF) [45]. It has been suggested that TAVI, often with a SE prosthesis, is a viable choice in this situation. Prosthesis migration and PVL can be prevented by ensuring appropriate prosthesis oversizing in the absence of valvular calcification. The size of the existing SE prosthesis might prove insufficient for aortic annulus anatomy, requiring significant oversizing in order to fit without calcification. The first case of a patient with LVAD-related AR receiving a 32 mm BE Myval prosthesis was documented by Ancona et al. [46]. The study concluded that a large BE prosthesis can be considered when significant oversizing is needed.

### 8.8. Myval via Trans-Carotid Access and Trans-Axillary Route in Patients with PVD

Recent research indicates that 10% to 15% of individuals are still not suitable candidates for transfemoral access. Trans-subclavian-axillary or trans-carotid access, as opposed to transapical and direct aortic access, has become more prevalent in patients with inappropriate femoral routes [47]. Bypassing the difficulties of the aorta-iliofemoral arteries, the trans-carotid route provides a relatively straight conduit from the carotid artery to the aortic valve. Trans-carotid TAVI (TC-TAVI) with BE Myval THV system is used in patients with a prohibitive abdominal aortic disease. Ayhan et al. and Keleş et al. published the paper on the first trans-axillary artery route experience with Myval implantation in Turkey [48,49]. No short-term complications via carotid artery access were reported.

### 8.9. ViV with Myval THV

The transcatheter ViV technique is now considered the first-line therapy for failed bioprosthetic heart valve (BHV) in patients deemed unsuitable for conventional redo surgery. Despite being effective and safe, the ViV procedure has been associated with more challenges compared to TAVI [50]. Ielasi et al. published a study of four patients who underwent aortic and one who underwent mitral ViV implantation [50]. Device success was achieved in all five presented cases. ViV in a stentless full root bioprosthesis is a risk factor for early (ECO) and delayed coronary obstruction (DCO) [51]. Recent evidence demonstrates that ViV procedures with a BE-THV, compared to SE ones, are associated with a lower DCO risk when coronary protection is managed by stent implantation compared to the “wire-only” strategy.

Despite a significant surgical risk, transcatheter ViV/ViR implantation for failing left side cardiac bioprosthesis can be successfully carried out using the Myval THV with a high success rate and low early and mid-term mortality and morbidity. This was demonstrated in a study done by Moscarella et al. The Myval THV was used for the transcatheter implantation of aortic ViV and mitral ViV/ViR in 97 consecutive patients who had symptomatic, severe aortic (*n* = 33) and mitral (*n* = 64) BHVs/ring dysfunction [52]. Ninety-five patients (98%) had technical success. Both aortic and mitral ViV/ViR implantation resulted in a considerable decrease in prosthetic transvalvular pressure gradients and an increase in valve areas after 30 days. At 15 months, the overall survival rate was 92%.

#### 8.9.1. Transcatheter Mitral Valve-in-Valve Implantation (ViV) with Myval THV

Transseptal ViV mitral implantation (TMViV) using BE Myval THV has proven to be a safe and practical means of preventing recurrent surgery in high-risk patients with bioprosthesis degeneration. Transseptal and transapical approaches have been used for replacement for degenerated THVs using TMViV and have been described [53]. Blasco-Turrión et al., in 2022, conducted a multicenter retrospective study of 11 high-risk surgical patients across five institutions with mitral bioprosthesis degeneration undergoing transcatheter ViV implantation with Myval THV [54]. The peak and mean transvalvular gradients were 27 ± 5 mmHg and 14.7 ± 2.3 mmHg, respectively. The predicted neo-left ventricular outflow tract (neo-LVOT) area was 183.4 ± 56 mm^2^ (range: 171 to 221 mm^2^). The procedures were performed via transfemoral access in all cases. Overall, 100% technical success was achieved, with no significant residual mitral stenosis (peak 7.2 ± 2.7 and mean gradient 3.4 ± 1.7 mmHg) and no complications during the procedure. There were no cases of LVOT obstruction, migration, or PVL. At a 6-month follow-up, there was one incidence of inadequate anticoagulation with an elevation in transmitral gradients (mean 15 mmHg) that reversed following anticoagulation adjustment, but there were no other noteworthy occurrences.

Similar results were demonstrated in a retrospective study done by Sankardas et al. [55]. TMViV replacement with Myval was safely performed with high technical success and low 30-day and 1-year mortality. In this study, 20 symptomatic patients with surgical bioprosthetic mitral valve failure with New York Heart Association (NYHA) class III–IV symptoms, despite optimal medical therapy and high or very high risk for redo surgery, were assigned to TMViV. In 60% of patients, a combination of stenosis and regurgitation was the cause of failure. In all cases, technical success was achieved. After the intervention, the mean gradient was 4.6 ± 2.7, and after 30 days, it was 6.3 ± 2.1. There were no discernible left ventricular outflow tract obstructions or substantial valvular or paravalvular leakage. At one year, 10% of deaths were due to all causes, and all survivors belonged to NYHA classes I or II.

#### 8.9.2. Myval in Tricuspid ViV

Valve dysfunction/degeneration after tricuspid valve replacement or repair utilizing an annuloplasty ring is common [56]. It increases morbidity and mortality of the patients. Currently, to overcome this dilemma, the transcatheter tricuspid valve-in-valve (TVIV) and valve-in-ring (TVIR) implantation procedures have emerged as important alternatives for high-risk patients. Ayhan et al. published the first of its kind case report demonstrating that the BE Myval THV system is suitable for transcatheter TVIR [48]. Recently, Mussayev et al. demonstrated the BE Myval THV to be safe and effective, on par with BE Sapien 3 and SE Evolut R THVs [57]. The case series consists of five unique cases of high-risk patients with prior surgeries, with clinically indicated tricuspid ViV procedures. Using the BE Myval THV, transcatheter tricuspid ViV implantation achieved good immediate and one-year clinical results that were safe and feasible. The results of this investigation indicated that in comparison to other commercially available THVs, the larger range of appropriate THV sizes may be associated with the new BE Myval THV’s superior performance. The intermediate sizes, in particular, mitigate the risks of both oversized or incomplete ViV “sealing” associated with free wall rupture, significant PVL or premature THV dysfunction.

### 8.10. Myval for Pulmonary Valve Replacement

After surgery for CHDs like Rastelli, Ross, and complete repair of Tetralogy of Fallot, pulmonary regurgitation and pulmonary stenosis are major causes of morbidity. For the past 20 years, percutaneous pulmonary valve implantation (PPVI) has been used as a less intrusive substitute for surgery [58]. Replacing the pulmonary valve in individuals with congenital heart conditions and heart failure presents significant challenges. The Myval delivery system offers significant advantages for PPVI [59]. With a 14 Fr introducer sheath for the 29 mm valve, it allows easier advancement through the right ventricular outflow tract compared to the 16 Fr sheath needed for the 29 mm Edwards S3 valve and the 22 Fr sheath for the 24 mm Melody valve. Its crimping on the balloon catheter enhances deployment in complex anatomical settings. The option of a 32 mm valve is particularly beneficial for larger RVOT, broadening the patient’s eligibility for treatment. The Myval THV’s unique “dog bone” configuration during inflation aids precise positioning, especially in valve-in-valve procedures. Follow-up results show functional implanted valves with no PVL, likely due to the external PET skirt that reduces this risk. Overall, this study highlights Myval as a clinically feasible, safe, and effective option for pulmonary interventions, with further follow-up needed for long-term efficacy assessment.

Odemis and Yenidogan published a case series of nine patients, aged 8 to 34 years, who underwent Myval implantation in the pulmonary position between June and November 2020 [59]. Diagnoses included Tetralogy of Fallot and pulmonary stenosis. Stent implantation preceded valve placement, with sizes ranging from 23 mm to 29 mm. No valvular leakage was detected immediately after the procedure. Follow-up evaluations showed competent valve function and no dysrhythmias, with a mean follow-up of 9.8 months.

### 8.11. Myval for Aortic Stenosis and Cardiac Amyloidosis

A major gap remains in evaluating Myval’s performance in patients with both aortic stenosis and cardiac amyloidosis, an increasingly recognized condition where THV therapy is particularly challenging due to the infiltrative nature of the disease. In these patients, cardiac amyloidosis can complicate valve implantation, impacting procedural success and long-term outcomes. Further research is essential to assess Myval’s applicability in this complex patient group, as well as to examine long-term durability and outcomes across diverse populations [60].

## 9. Importance of Extra-Large (XL) Size of Myval THV

Sathananthan et al., in their study, reported mechanical valve dysfunction in some Sapien 3 prostheses after bench-testing overexpansion [61]. Overexpansion was examined in a limited number of prosthesis cases with up to 3 mL of extra volume in this bench test. The authors draw the conclusion that severe overexpansion may be linked to acute leaflet failure, decreased durability, and compromised hydrodynamic performance. The ex vivo studies escalated apprehension of long-term durability of over-expanded THV with regard to damage of tissue in the bioleaflets. Sellers et al. performed an ex vivo study to determine the impact of overexpansion on leaflet ultrastructure across different valve sizes (23, 26, and 29 mm) [62]. Leaflet thinning, increased tissue density within the leaflet matrix, and a significant increase in the entropy of fibrillar collagen (on both the aortic and ventricular aspect of the leaflets) were indicators of ultrastructure damage to the leaflets that the authors observed in the overexpanded valves when compared to the nominally expanded control valves. More long-term follow-ups are of key importance for future successful implementation.

The Myval THV series offers a wide range of THV sizes, including traditional sizes (20, 23, 26, and 29 mm), intermediate sizes (21.5, 24.5, and 27.5 mm), and extra-large sizes (30.5 and 32 mm). The expanded size options in the Myval THV portfolio, particularly at the larger end, may effectively address concerns regarding size limitations for most patients. Currently, the largest standard devices on the market, such as the Sapien 3 from Edwards, can accommodate an annular area of 683 mm^2^, while the Evolut R from Medtronic has an annular perimeter limit of 94.2 mm according to the manufacturer’s instructions. To tackle the issue of size limitations in existing THV products, the Myval THV series has introduced a diverse range of sizes, including XL options of 30.5 mm and 32 mm. Notably, the 32 mm Myval THV is the largest aortic THV available and is designed to cover annular areas ranging from 700 to 840 mm^2^. This device is specifically intended for use in aortic annuli measuring up to 840 mm^2^.

### 9.1. The 32 mm Myval for Treatment in Patients with Extremely Large Aortic Annuli in Real-World Scenario: First Global, Multicenter Experience

In a retrospective study by Holzamer et al., data were collected from 10 patients implanted with 32 mm Myval THVs who had AS and very large annular anatomy (mean area 765.5 mm^2^) from eight centers [63]. VARC-2 device success was achieved in all cases. Mild PVL was observed in three patients, and two patients required new PPI. Three patients had mild PVL, and two of them needed replacement pacemaker implantations. Only one patient needed surgical revision because of retroperitoneal bleeding due to contralateral 6 Fr sheath. There were no complications linked to the device, strokes, or deaths from any cause throughout the 30-day follow-up period. Only 0.27% of patients in a sample of 2219 consecutive TAVR-screened patients from a central European site had anatomy greater than that allowed by the 32 mm Myval device according to use guidelines without off-label overexpansion. Rates were higher for 34 mm Evolut Pro (1.8%) and 29 mm Sapien 3 (2.1%) devices. Promising preliminary outcomes were observed with the Myval 32 mm prosthesis in a group of patients who had previously been denied TAVI. In order to treat every patient as effectively as possible, it is ideal for all future TAVI systems to support bigger anatomy. The nested XL registry of the LANDMARK trial, which is actively recruiting patients, is the first prospectively collected and managed trial cohort that includes individuals with extra-large anatomy. The trial’s data should provide some evidence in this area.

### 9.2. XL-Myval 32 mm in Large Bicuspid Anatomy

BAV is considered an unfavorable anatomy for TAVI due to its exclusion from randomized trials. The relatively large aortic root architecture, including annulus diameters, that may surpass the size matrix of the currently available commercial TAVI devices, along with the concomitant high calcium burden, fused raphe, and aortic dilatation, is a key challenge within BAV [64]. Elkoumy et al. reported BAV to be safe with promising and acceptable hemodynamic and clinical outcomes in a case series of three patients with low surgical risk and severe calcific BAV stenosis with large annular dimensions, treated with the novel BE Myval-XL 32 mm valve [65].

A case report of a PVR with 32 mm Myval has been reported in native RVOT in a patient with surgically corrected Tetralogy of Fallot with good results [66].

## 10. Clinical Advantages and Design Innovations of the Myval Transcatheter Heart Valve

The Myval THV series offers specific advantages that may make it preferable in certain clinical scenarios, particularly due to its unique size offerings, including intermediate (21.5 mm, 24.5 mm, and 27.5 mm) and extra-large (30.5 mm and 32 mm) sizes. These options allow for precise matching to patient anatomies, especially those with large annuli or annuli with anomalous morphology, helping to avoid the common issues of under-sizing or oversizing seen with other THVs. Clinical evidence indicates that Myval THVs are associated with lower rates of PVL and reduced PPI compared to some SEVs, especially in high-risk and anatomically complex cases. Additionally, for patients with large annuli, Myval’s larger sizes are particularly beneficial, minimizing adverse events related to overexpansion [29].

## 11. Gaps in Evidence and Future Directions

The Myval THV demonstrates promising results across various patient anatomies, but further investigation is necessary. Key gaps include the need for larger, randomized trials to compare Myval with other THVs in diverse populations. While short-term data is encouraging, its long-term durability, particularly for newer models and larger sizes, needs verification. Although Myval’s range of intermediate sizes enables precise anatomical matching, the clinical impact of this tailored approach is yet to be proven. Future studies should assess Myval’s extended performance, refine sizing methods, and evaluate its suitability for broader applications, such as small annuli and bicuspid valves, to build stronger evidence-based guidelines for its application.

## 12. Conclusions

Compared to the previous generation of balloon-expandable THVs, Myval is a technical development that makes the procedure safer, easier, and perhaps even more successful. The device’s efficiency and long-term durability are being compared to contemporary self-expandable and balloon-expandable THVs in large prospective randomized trials. With the ongoing large, randomized LANDMARK trial, comparing its safety and effectiveness to contemporary self-expandable and balloon-expandable devices, Myval has proven its non-inferiority in terms of early outcomes. If Myval is proven to be long-term non-inferior, it may make TAVI more accessible and affordable for a far greater number of patients worldwide. Another randomized trial, Compare-TAVI, is ongoing with comparison between Myval and Sapien, and its results are awaited. While early outcomes are promising, particularly in high-risk patients, the conclusions are limited by small sample sizes. Further research, including larger multicenter studies, is essential to confirm these findings and assess long-term efficacy across various risk categories.

## Figures and Tables

**Table 1:** Indications for aortic valve replacement in aortic stenosis [2].

S. No.	Recommendations	Class of Recommendation/Level of Evidence
1	In adults with severe high-gradient AS (Stage D1) and symptoms of exertional dyspnea, HF, angina, syncope, or presyncope by history or on exercise testing	Class 1/A
2	In asymptomatic patients with severe AS and an LVEF < 50% (Stage C2)	Class 1/B-NR
3	In asymptomatic patients with severe AS (Stage C1) who are undergoing cardiac surgery for other indications	Class 1/B-NR
4	In symptomatic patients with low-flow, low-gradient severe AS with reduced LVEF (Stage D2)	Class 1/B-NR
5	In symptomatic patients with low-flow, low-gradient severe AS with normal LVEF (Stage D3), if AS is the most likely cause of symptoms	Class 1/B-NR
6	In apparently asymptomatic patients with severe AS (Stage C1) and low surgical risk, AVR is reasonable when an exercise test demonstrates decreased exercise tolerance (normalized for age and sex) or a fall in systolic blood pressure of ≥10 mm Hg from baseline to peak exercise	Class 2a/B-NR
7	In asymptomatic patients with very severe AS (defined as an aortic velocity of ≥5 m/s) and low surgical risk, AVR is reasonable	Class 2a/B-R
8	In apparently asymptomatic patients with severe AS (Stage C1) and low surgical risk, AVR is reasonable when the serum B-type natriuretic peptide (BNP) level is >3 times normal	Class 2a/B-NR
9	In asymptomatic patients with high-gradient severe AS (Stage C1) and low surgical risk, AVR is reasonable when serial testing shows an increase in aortic velocity ≥ 0.3 m/s per year	Class 2a/B-NR
10	In asymptomatic patients with severe high gradient AS (Stage C1) and a progressive decrease in LVEF on at least three serial imaging studies to <60%, AVR may be considered	Class 2b/B-NR
11	In patients with moderate AS (Stage B) who are undergoing cardiac surgery for other indications, AVR may be considered	Class 2b/C-EO

**Class of recommendation:** Class 1: Strong recommendation; Class 2a: Moderate recommendation; Class 2b: Weak recommendation; **Level of evidence—Level A:** High-quality evidence from more than one randomized controlled trial (RCT); meta-analyses of high-quality RCTs; one or more RCTs corroborated by high-quality registry studies; **Level B-R:** Moderate-quality evidence from one or more RCTs; meta-analyses of moderate-quality RCTs; **Level B-NR:** Moderate-quality evidence from one or more well designed, well-executed non-RCT studies, observational, or registry studies; meta-analyses of such studies; **Level C-EO:** Consensus of expert opinion based on clinical experience; AS: aortic stenosis; AVR: aortic valve replacement; BNP: B-type natriuretic peptide; HF: heart failure; LVEF: left ventricular ejection fraction.

**Table 2:** Recommendations for aortic valve replacement in aortic stenosis by intervention type: TAVI vs. SAVR [2].

Class of Recommendation/Level of Evidence	SAVR	TAVI	Shared Decision-Making
Class 1/A	For symptomatic and asymptomatic patients with severe AS and any indication for AVR who are <65 years of age or have a life expectancy > 20 years	For symptomatic patients with severe AS who are >80 years of age or for younger patients with a life expectancy < 10 years and no anatomic contraindication to transfemoral TAVI, transfemoral TAVI is recommended	For symptomatic patients with severe AS who are 65 to 80 years of age and have no anatomic contraindication to transfemoral TAVI, either SAVR or transfemoral TAVI is recommended after shared decision-making about the balance between expected patient longevity and valve durability
Class 1/B-NR	For asymptomatic patients with severe AS and an abnormal exercise test, very severe AS, rapid progression, or an elevated BNP (COR 2a indications for AVR)	-	In asymptomatic patients with severe AS and an LVEF < 50% who are ≤80 years of age and have no anatomic contraindication to transfemoral TAVI, the decision between TAVI and SAVR should follow the same recommendations as for symptomatic patients in three recommendations given above
Class 1/A	For patients with an indication for AVR for whom a bioprosthetic valve is preferred but valve or vascular anatomy or other factors are not suitable for transfemoral TAVI	For symptomatic patients of any age with severe AS and a high or prohibitive surgical risk, TAVI is recommended if predicted post-TAVI survival is >12 months with an acceptable quality of life	-
Class 1/C-EO	-	-	For symptomatic patients with severe AS for whom predicted post-TAVI or post-SAVR survival is <12 months or for whom minimal improvement in quality of life is expected, palliative care is recommended after shared decision-making, including discussion of patient preferences and values

**Level of evidence—Level A:** High-quality evidence from more than one randomized controlled trial (RCT); meta-analyses of high-quality RCTs; one or more RCTs corroborated by high-quality registry studies; **Level B-NR:** Moderate-quality evidence from one or more non-RCT, observational, or registry studies; meta-analyses of such studies; **Level C-EO:** Consensus of expert opinion based on clinical experience. AS: Aortic stenosis; AVR: aortic valve replacement; BNP: B-type natriuretic peptide; COR: class of recommendation; LVEF: left ventricular ejection fraction; SAVR: surgical aortic valve replacement; TAVI: transcatheter aortic valve replacement.

**Table 3:** Early (30-day) clinical and hemodynamic outcomes of Myval in comparison to other THVs.

S. No.	Study	THV Comparison	Patient Population	Clinical Outcomes	Hemodynamic Outcomes
1	Kawashima et al. [22]	Myval (*n* = 108) vs. Sapien 3 (*n* = 397) vs. Sapien XT (*n* = 239)	AR	Myval showed lower PVL attributed to its external skirt design and intermediate sizes	Myval THV had the lowest rate of moderate/severe quantitative AR (2.8%) compared to Sapien 3 (8.3%) and Sapien XT (10.9%)
2	Delgado-Arana et al. [24]	Myval (*n* = 103) vs. Sapien 3 (*n* = 103)	Severe symptomatic AS	30-day mortality: Myval (0.97%) vs. Sapien 3 (2.9%) (*p* = 0.096); Lower rate of PPI with Myval (5.8% vs. 15.5%; *p* = 0.02)	Lower mean gradients for Myval (*p* < 0.001); and comparable rates of ≥moderate AR [Myval (0%) vs. Sapien 3 (1%); *p* = 0.314]
3	Barki et al. (EVAL registry) [25]	Myval (*n* = 58) vs. Evolut R SEV (*n* = 108)	Severe symptomatic AS	All-cause mortality: Myval—5.2% vs. Evolut—12.3%; comparable disabling stroke between groups; lower PPI with Myval (11% vs. 27.5%; *p* = 0.02)	Lower incidence of moderate-severe PVL in Myval group (6.9% vs. 19.8%, *p* = 0.039)
4	Halim et al. [26]	Myval (*n* = 91) vs. Evolut THV (*n* = 91)	Severe symptomatic AS	Cardiac death (1% vs. 2%, *p* = 0.56); Stroke (2% vs. 4%, *p* = 0.41); MI (1% vs. 3%, *p* = 0.31), and PPI (4% vs. 15%, *p* = 0.01)	Aortic valve area (1.98 ± 0.5 cm^2^ vs. 2.13 ± 0.5 cm^2^; *p* = 0.08); Mean gradient (7.8 ± 3.2 mmHg vs. 7.6 ± 3.2 mmHg; *p* = 0.63); Moderate to severe PVL (1% vs. 4%; *p* = 0.17)
5	Baumbach et al. [29]	Myval (*n* = 384) vs. Contemporary valves (Sapien and Evolut series) (*n* = 384)	Severe symptomatic AS	Comparable rates of all-cause mortality, all stroke, bleeding (types 3 & 4), and major vascular complications between Myval and contemporary series. New PPI rates: 15% in Myval vs. 17% in contemporary series (*p* = 0.49).	Moderate or severe PVR: 3% vs. 5%; *p* = 0.58 (Myval vs. contemporary valves). Mean gradient: Myval—8.20 mmHg vs. Contemporary—7.9 mmHg; *p* = NS. Effective orifice area: Myval—2.02 cm^2^ vs. Contemporary—2.05 cm^2^; *p* = NS.

AS: Aortic stenosis; AR: Aortic regurgitation; PVL: Paravalvular leak.

## Data Availability

No new data were created or analyzed in this study. Data sharing is not applicable to this article.
